# Dastardly Outrage upon a Physician

**Published:** 1891-03

**Authors:** 


### A DASTARDLY OUTRAGE ON A PHYSICIAN.

The JOURNAL blushes to record the occurrence in a civilized community of such an outrage as that perpetrated by Oscar Samostz, a Bohemian druggist of Austin, upon Dr. T. J. Bennett, one of the leading physicians of Austin, and a most exemplary gentleman.

Drs. Bennett and Morris had for years been occupying an office over Samostz' store, and on leaving there to occupy their new and elegant quarters, some feeling must have been engendered on the part of the irascible little foreigner, and a misunderstanding occurring between him and Dr. B. as to the removal of certain equipments which Drs. Bennett and Morris had put in the office, he took occasion to vent his spite by attempting, as is alleged in the indictment, to put Bennett's eyes out with sulphuric acid.

The testimony of Dr. Bennett and Mr. Burke, the plumber, who had accompanied him to remove the fixtures, is, that Dr. Bennett, seeing Samostz' design, as he came toward him with the glass raised in a threatening manner, endeavored to thwart his fiendish purpose by catching his arm. He arrested it just as the contents of the glass were dashed at his face; but ducking his head just in time, the charge was received on the brim of his handsome spring "stovepipe" hat, excepting a few drops which struck his face just under and to the side of his left eye. (The "stovepipe," it is unnecessary to say, was demolished.) The acid on the face caused a painful and severe burn, and had it gone into the eye, would surely have destroyed both eye and sight. The Doctor gave his antagonist a sound drubbing with his fist before he felt the burning of the acid. He then hastened to apply an alkali and wash the wounds, and to-day is not the least disfigured; but it was a close call.

Samostz was indicted by the grand jury on three counts: maiming, difiguring, and assault. The trial, which took place recently, (about March 6-8), resulted in a compromise verdict, giving him the lowest penalty allowed by law,—two years in the county jail. It is currently reported that eleven jurors were for five years in the penitentiary, the maximum,— and one, a colored man, the only one on the jury, held out for the minimum penalty, and hung the jury for more than twenty-four hours. Samostz was promptly jailed, and his attorneys moved for a new trial, but it was overruled; they then took an appeal, and this man who fights with an outlawed and barbarous weapon, is out on bail. Reputable citizens allege that he has been known to throw sulphuric acid on the legs of horses which have been hitched in front of his store. Is this the nineteenth century? and can such things be, and be tolerated by a civilized and peace-loving community? Heretofore Samostz has been one of the leading druggists, and was patronized by the best people of the city and county. His store, on the Avenue, was one of the handsomest and best equipped pharmacy establishments in the South; and a career of success and prosperity seemed to be opened up for the proprietor; but he has in a fit of anger thrown away his opportunity and alienated many who gave him a strong patronage and support.

A man who would with malice aforethought attempt to put out the eyes of a fellow-creature, deserves the severest penalty of the law; but what punishment is severe enough for a human fiend, who would throw acid on a dumb brute? a horse—man's faithful friend and servant?

Samostz' plea was "self-defense," and his shrewd lawyer worked it for all it was worth, doubtless saving him from the severest penalty of the law. He claimed that after handling aniline dyes, he was in the habit of washing his hands with sulphuric acid, and that on this occasion, his hands being very much soiled, he had poured out the acid for the purpose of cleansing them, preparatory to going on the street. It looks like an impossibility to pour commercial sulphuric acid in the palms of the hand without great pain and burning; but he was asked to demonstrate his procedure to the jury, and he did so without flinching. A quantity of acid had been procured from another drug store, and Samostz, after blackening his hands with a dye, deliberately poured a dram or two of acid in his palm and laved his hands in it thoroughly, all over; he then dipped them in a basin of water and it cleansed them of the stain. This made a strong impression on the jury, and caused them to entertain some doubt as to malice, else he would surely have fared worse. Just how he did it, without injury to his hands—he must be a Salamander—is "one of those things" we cannot find out. We do not think an ordinary man could do it; we should not like to try it. An acid that will burn a hole in a stovepipe hat, is one of the things that *we* would not like to handle. But this "extraordinary citizen of Austin" treated it with the intimacy—almost fondness—of a tender parent!

